# Reliability and construct validity of the Early Dementia Questionnaire (EDQ)

**DOI:** 10.1186/s12877-016-0384-1

**Published:** 2016-11-30

**Authors:** Zurraini Arabi, Syed Alwi Syed Abdul Rahman, Helmy Hazmi, Nazeefah Hamdin

**Affiliations:** 1 Department of Family Medicine, Faculty of Medicine and Health Sciences, Universiti Malaysia Sarawak, Kota Samarahan, Sarawak 94300 Malaysia; 2 Department of Community Medicine and Public Health, Faculty of Medicine and Health Sciences, Universiti Malaysia Sarawak, Kota Samarahan, 94300 Sarawak Malaysia

**Keywords:** Dementia, Early Dementia Questionnaire, Primary care, Screening

## Abstract

**Background:**

Early Dementia Questionnaire (EDQ) was developed as a screening tool to detect patients with early dementia in primary care. It was developed based on 20 symptoms of dementia. From a preliminary study, EDQ had been shown to be a promising alternative for screening of early dementia. This study was done to further test on EDQ’s reliability and validity.

**Methods:**

Using a systematic random sampling, 200 elderly patients attending primary health care centers in Kuching, Sarawak had consented to participate in the study and were administered the EDQ. Geriatric Depression Scale (GDS) was used to exclude patients with depression. Those who scored >21 MMSE, were retested using the EDQ. Reliability was determined by Cronbach’s alpha for internal consistency and construct validity was assessed using confirmatory factor analysis (principle component with varimax rotation). Test retest Intraclass Correlation Coeeficient (ICC) was used to determine the reliability of the scale.

**Results:**

The result showed that the sensitivity and specificity for EDQ was 71.2% and 59.5%. The overall Cronbach’s alpha coefficient was good which was 0.874. Confirmatory factor analysis on 4 factors indicated that the Cronbach’s alpha for each domain were acceptable with memory (0.741), concentration (0.764), emotional and physical symptoms (0.754) and lastly sleep and environment (0.720). The Intraclass Correlation Coefficient between the first EDQ score and the retest EDQ score among those with MMSE of >21 showed a very strong overall agreement, ICC = 0.764, *N* = 160, *P* <0.001.

**Conclusions:**

The results of the validation study showed that Early Dementia Questionnaire (EDQ) is a valid and reliable tool to be used as a screening tool to detect early dementia in primary care.

## Background

The number of ageing population is increasing globally with the expected increase of those aged 60 and above to 2 billion by the year 2050 [1]. In 2008, the proportion of those aged 60 years and above among Malaysians was 7% or a total of 1.9 million elderly persons [2]. This percentage is expected to increase to 9.8% in year 2020 [3]. One of the common age related illness is dementia. In 2000, more than 25 million people had suffered from dementia and the number is expected to increase in year 2030 to 63 million people [4]. Thus, as the population ages, the prevalence of dementia will also increase. Dementia is defined as progressive and largely irreversible clinical syndrome which is characterized as global impairment of mental function [5].

The rational of identifying dementia in its early stages is it enables early intervention process. This will allow appropriate treatment to be initiated on time, delay the consequences of dementia and for patients to maintain their daily life functions for as long as possible. Timely diagnosis will allow the person with dementia and family members to make choices about their future care while they are still able, plan their finances and legal matters while decision making capacity remains and to access medications that may relieve symptoms [6, 7]. It gives time for the carers to adjust with the sequelae of dementia and prepare options for long term care.

There are many tools available used to detect dementia in primary care. This includes the Mini Mental State Examination (MMSE) which is acknowledged as the gold standard for cognitive screening. It has been translated in many languages to be used in many countries worldwide. Even though MMSE had been used widely to assess for cognitive impairment, it has its own limitations and weaknesses. MMSE is lacking in its sensitivity towards the early sign of dementia and presents a “ceiling effect” that results to a false-negative diagnosis where those with normal MMSE score may still have cognitive impairment [8]. MMSE does not have questions that assess function such as testing capacity to abstract or to judge a social situation which consequently cause difficulty in early detection of dementia. Factors such as study settings, patient’s educational and cultural background are also known to affect the findings.

In Malaysia, MMSE is one of the commonest screening tools used in primary care clinics and hospital setting in patients with suspected cognitive impairments [9]. The English version of MMSE was found not suitable due to language barriers and cultural differences. In order to be used in our community, the Malay version of MMSE has been translated and validated by Norlinah et al. (2009). The optimal cut-off score were 21 (sensitivity 88.5, specificity 75.3), 18 (sensitivity 97.1, specificity 90.0) and 17(sensitivity 97.7, specificity 93.3) [10] However, despite its limitations, it is still the most studied and often used as reference for comparative studies of other assessment tools in detection of cognitive impairment and dementia [11]. With these reasons, MMSE was used as the gold standard in this study.

Most screening tools available concentrates on cognitive functions and requires training of the person conducting the test to ensure standardization of administration and scoring. This presents as barriers to widespread use of these screening tools in primary care setting especially in rural areas. Patient’s cultural and educational background also limits the use of currently available tools in detecting cognitive impairments. Thus, Early Dementia Questionnaire (EDQ) was developed to screen elderly patients for early dementia in primary care based on symptoms of dementia to overcome these barriers. A preliminary study using this newly developed questionnaire showed that comparing to MMSE, it was a simpler, easier to be administered in a shorter time and a user friendly tool where it can be used in a busy clinic. It is not fully dependent on patient, where the information could be obtained from the informant for scoring of the tool. It was also less influenced by educational level and patient’s social or cultural background [12]. This study was done to further validate the EDQ to assess its reliability and construct validity. Although a cut-off of 8 was used in the preliminary study, finding the suitable cut-off based on the current validation study is important to increase the accuracy of EDQ in detecting early dementia.

## Methods

### Sample population

This was a cross-sectional study involving elderly patients aged 60 and above attending primary health care clinic in Kuching, Sarawak, Malaysia. Respondents were recruited from March till July 2014 using systematic random sampling. Respondents chosen were well adults who came to the clinic for follow ups of their current medical illness. However, those who are already diagnosed with dementia or depression, aphasic and had severe hearing impairment were excluded from the study. Eligible respondents and their informants, who came together to the clinic, were explained about the study rationale and informed consents were taken before they attempted the questionnaire. Informants were either their spouse or adult child.

From the preliminary assessment, eligible respondents were screened for possible underlying depression using the Geriatric Depression Scale (GDS). Those who scored 5 and below, were included in the study. Those who have possible depression were excluded from the study and were managed accordingly.

A total of 200 respondents were recruited in the study. Sociodemographic information such as age, gender, race, income per month, marital status, educational level and their current medical illness were obtained from the respondents. Subsequently, a face to face interview using both the MMSE and the EDQ was done by trained researchers with the respondents to ensure the right answers and appropriate explanations were given if the respondents or informants had doubt or unclear about the questionnaires. Besides the respondents, the respondents’ informants were also interviewed using a separate EDQ to corroborate the responses of the respondents. Informants who were not with the respondents in the clinic were interviewed via phone at a later period (within 1 week). Respondents who scored >21 on the MMSE (160 respondents) were considered as not having dementia, were followed up and retested with the EDQ five months later. Prior to the retesting with the EDQ standard history and physical examination was done to ensure no deterioration in their health status.

### Assessment tools

#### Geriatric Depression Scale (GDS)

The validated Malay Geriatric Depression Scale-14 (M-GDS-14) [13] was used in this study as a tool to rule out pseudodementia due to depression before subjects were tested with EDQ. A cutoff point of 6 was used to detect depression with 95.5% sensitivity and 84.2% specificity [14].

#### Early Dementia Questionnaire (EDQ)

Early Dementia Questionnaire was developed based on combination of literature review, the expert opinion of Family Medicine Consultants and Geriatric Psychiatrists and a standard assessment tool [12]. It is an interviewer guided questionnaire answered by both patient and their informant. The EDQ contains 20 items based on the symptoms of early dementia.

Symptoms of dementia were divided into 6 sub-domains; memory symptoms, concentration, physical symptoms, emotions, sleep disturbance and others. Memory symptoms (5 questions) were checklist as memory support, difficulty in remembering events happening in the past 1 week (recent memory), unable to find kept/stored items and difficulty in remembering names/ familiar faces and familiar road directions. Concentration symptoms (4 questions) were difficulty in following conversation, difficulty in understanding reading, difficulty in following stories in television and repetitive questioning. Physical symptoms (3 questions) were difficulty carrying out daily house chores / work / hobby, difficulty in taking care of self / personal hygiene or using the toilet and disrupted movement (physical restlessness). Emotional symptoms (4 questions) were unsuitable reaction towards external stimuli, obsession towards emotional events which has happen a long time, apathy/ no passion or not interested in surrounding and looking for support/assurance from partner. Sleep disturbance (2 questions) were night-day sleep rhythm disruption and restlessness at night. Other symptoms (2 questions) were confusion after moving houses / in a new environment and outsiders aware of changes in term of behavior / appearance.

The scoring for each symptoms ranged from 0–3 with 0 as never, 1 seldom, 2 sometimes and 3 always. These were based on the symptoms the patient had in a week for the past 2 years. The minimum score was 0 and maximum score was 60. If there was any discrepancy between the respondens’ and informant’s response, the response with the higher score for each symptom will be taken for total score. Following the preliminary study, a score of 8 or more indicates that a patient had possible early dementia.

#### Mini Mental State Examination (MMSE)

The original version was developed by Folstein et al in 1975. The MMSE is still being widely used as a screening tool in dementia. In Malaysia, the Malay version of MMSE has been translated from the original version and validated by Norlinah et al (2009) [10]. The validated Malay version of MMSE-7 (serial 7) was used for this validation study with the cut-off level of 21 and below used to detect possible dementia.

### Statistical analysis

Data analysis was done using the Statistical Package for the Social Sciences (SPSS) for windows (Version 20). Descriptive analysis was used for the sociodemographic data. Reliability was determined by Cronbach’s alpha for internal consistency and construct validity was assessed using confirmatory factor analysis (principle component with varimax rotation). For test retest reliability, intraclass correlation coefficient (ICC) was used, ranging from one (perfect) to zero.

## Results

### Sociodemographic characteristics

A total of 200 elderly patients were included in the study. Ninety six (48%) of the patients were male while hundred and four (52%) were females with the mean age of 68.5 years. Ninety five (47.5%) of the informants were interviewed by face to face while 105 (52.5%) were interviewed by phone. Only 25 (12.5%) of the patients and informant had similar score on EDQ. While 175 (87.5%) had a slightly different scores. Majority of them were Malays (73%) and married (74.5%). The sociodemographic characteristics of the study population were summarized in Table 1.

**Table 1:** Socio-demographic characteristics of the respondents (*N* = 200)

Variables	Mean (SD)	Median (IQR)	N	Percentage, %
Age	68.5 (6.28)			
Gender				
Male			96 (48.0)	
Female			104 (52.0)	
Race				
Malay			146	73
Chinese			24	12
Iban			16	8
Bidayuh			10	5
Melanau			2	1
Others			2	1
Monthly income (RM)		600.0 (1000.00)^a^		
Marital status				
Single			9	4.5
Married			149	74.5
Divorced/widowed			42	21.0
Educational level				
No formal education			59	29.5
Primary education			86	43.0
Secondary education			47	23.5
Tertiary education			8	4.0
Medical illness				
Hypertension				
No			51	25.5
Yes			149	74.5
Diabetes Mellitus				
No			113	56.5
Yes			87	43.5
Hypercholesterolemia				
No			96	48.0
Yes			104	52.0
Stroke				
No			197	98.5
Yes			3	1.5
Hypothyroid				
No			195	97.5
Yes			5	2.5
Heart Disease				
No			183	91.5
Yes			17	8.5

*SD* standard deviation, *IQR* Interquartile range, *N* frequency; ^a^The distribution is skewed to the right

### Prevalence of dementia

**Fig. 1:**
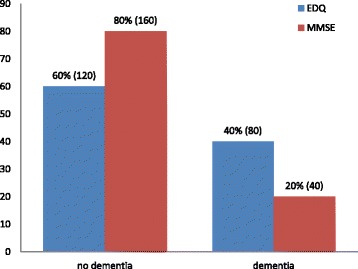
Prevalence of dementia using EDQ and MMSE

**Fig. 2:**
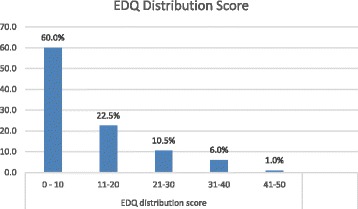
Distribution of EDQ score

**Fig. 3:**
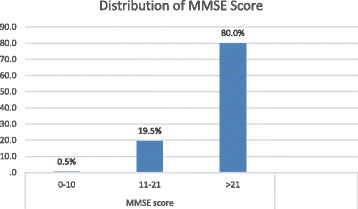
Distribution of MMSE score

The prevalence of early dementia using the newly designed EDQ in this study was 40.0% (95% CI 0.889, 2.787; *P* = 0.120), whilst the prevalence of dementia using MMSE was 20.0% (95% CI 5.065, 57.857 *P* < 0.001) as shown in Fig. 1. However, as this was a validation study, the number of study population was insufficient to provide an accurate prevalence which needs a larger sample size. The results of scoring for EDQ and MMSE in the study population were shown in Figs. 2 and 3 respectively.

### Scoring of EDQ

**Fig. 4:**
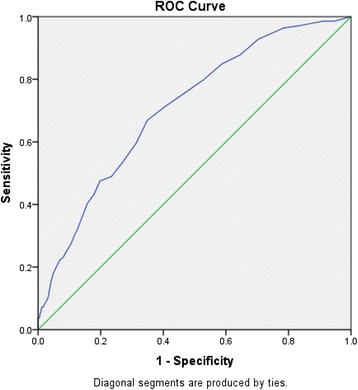
ROC curve in estimating the EDQ cut off point

In estimating the EDQ cut off point to the presence of dementia, the total EDQ score was plotted with the MMSE categorization using the ROC curve (Fig. 4). Suggestive cut off value for EDQ (generated from ROC curve) were shown in Table 2. The area under the ROC curve was 0.745 which showed that the EDQ score was able to accurately discriminate 74.5% of the cases. The best sensitivity and specificity was noted to be within the point of 7.5–10.5 in the EDQ range. A Kappa agreement analysis was conducted using the EDQ score cut off point of 8, 9 and 10 respectively with the reference to the MMSE categorization. Finally, the cut-off point of 10 was chosen based on the calculation of sensitivity and specificity which was 71.2% and 59.5% respectively as shown in Table 3.

**Table 2:** Suggestive cut off value for EDQ (generated from ROC curve)

EDQ score	Sensitivity	Specificity
7.5	0.799	0.471
8.5	0.755	0.533
9.5	0.712	0.595
10.5	0.669	0.651

**Table 3:** Sensitivity and specificity for different EDQ cut-off scores and Kappa value

Early Dementia Questionnaire (EDQ)		MMSE		Kappa value	Sensitivity	Specificity	P value for Kappa
		No	Yes				
EDQ 8	No	243	28	0.164	79.8%	47.1%	0.000
	Yes	273	111				
EDQ 9	No	275	34	0.187	75.5%	53.3%	0.000
	Yes	241	105				
EDQ 10	No	307	40	0.213	71.2%	59.5%	0.000
	Yes	209	99				

### Construct validity of EDQ

Although the questions in the original EDQ have been divided into 6 subdomains, exploratory factor analysis (EFA) was performed on all 20 symptoms and it extracted five factors. Multiple cross loadings were noted and one of the factors had less than 3 items. Despite extracting five factors, only two items load in one of the factors. A decision was made to extract four factors as shown in Table 6. The Kaiser-Meyer –Olkin (KMO) Measure of Sampling Adequacy was 0.864 (greater than 0.6) and the Bartlet’s test of Sphericity (BTS) was significant with *p* < 0.001, indicating that the factorability of the correlation matrix was assumed. The solution accounted for 53.2% of the variances with the first factor explaining the greatest amount of variance (19.5%).

A total of six items were loaded to component 1, three items to component 2, seven items to component 3 and the remaining 4 items were loaded to component 4. The components were named based on the associations of items within a component, retaining and combining some of the components from the original instrument.

From the original 6 subdomains of EDQ, the symptoms were rearranged and regrouped into 4 subdomains without omitting any of the questions. The symptom ‘repetitive questioning’ from the symptoms under concentration was noted to best fit into memory subdomain. Physical and emotional symptoms were grouped together (question 10–16) into one subdomain. The questions were; difficulty carrying out daily house chores/work/hobby, difficulty in taking care of personal hygiene or using the toilet, disrupted movement (physical restlessness), unsuitable reaction towards external stimuli, obsession towards emotional events which has happen a long time ago, not interested in surrounding and looking for support/assurance from partner. Questions 17–20 were also regrouped into one subdomain which was sleep and environment. The questions were; night-day sleep rhythm disruption, restlessness at night, confusion after moving houses / in a new environment and outsiders aware of changes in term of behavior / appearance. Thus, the final components were memory, concentration, physical/emotional and sleep/environment. Confirmatory factor analysis was done on these four subdomains (Table 4).

**Table 4:** Confirmatory factor analysis (CFA) on 4 factors using principle component with varimax rotation

Scale	Item	Loading on 4 factors			
		1	2	3	4
Memory	Require check list as memory support	0.493			
	Difficulty in remembering events that took place in the past 1 week (recent memory)	0.660			
	Unable to find kept item	0.641			0.343
	Difficulty in remembering names/familiar faces	0.743			
	Difficulty in remembering familiar road directions	0.522			
	Repetitive questioning	0.534			0.329
Concentration	Difficulty in following conversation		0.796		
	Difficulty understanding reading		0.777		
	Difficulty following stories on television		0.571	0.545	
Physical and emotional symptoms	Difficulty carrying out daily house chores/work/hobby			0.578	
	Difficulty in taking care/personal hygiene or using the toilet	0.396		0.349	
	Disrupted movement (physical restlessness)			0.707	
	Unsuitable reaction towards external stimuli (example: telephone ringing - emotional outburst)			0.700	
	Obsession towards emotional event, although it has taken place long time ago (example: death of family member or friend)			0.485	
	Not interested in surroundings			0.564	
	Looking for support/assurance from partner			0.620	
Sleep and environment	Night-day rhythm disruption				0.635
	Restlessness at night			0.422	0.662
	Confusion after moving houses/in a new environment				0.724
	Outsiders aware of changes in term of behavior/appearance				0.429

The overall factor loadings were noted to be in the range of 0.349–0.796. The “memory” component showed a loading range of 0.522–0.743. Two items in this component shared a cross loading with factor 4. The “concentration” component showed a range of loading between 0.571 and 0.796. “Physical and emotional symptom” component loading range were between 0.345 and 0.707 while “sleep and environment loading range were between 0.429 and 0.724. All three later components had at least one item cross loading into other components.

The convergent validity was determined by analysing the correlation between the items and the total score of the related construct as shown in Table 5. Because the variables were continuous, the Pearson correlation was chosen to test the correlation between the variable. The p-value was significant at 0.05.

**Table 5:** Correlation between items and items’ construct total score

Subdomain	Memory	Concentration	Physical and emotional symptoms	Sleep and environment
Item				
Require check list as memory support	0.565**			
Difficulty in remembering events that took place in the past 1 week (recent memory)	0.680**			
Unable to find kept item	0.708**			
Difficulty in remembering names/familiar faces	0.730**			
Difficulty in remembering familiar road directions	0.657**			
Repetitive questioning	0.514**			
Difficulty in following conversation		0.737^**^		
Difficulty understanding reading		0.804^**^		
Difficulty following stories on television		0.809^**^		
Difficulty carrying out daily house chores/work/hobby			0.620^**^	
Difficulty in taking care/personal hygiene or using the toilet			0.446^**^	
Disrupted movement (physical restlessness)			0.758^**^	
Unsuitable reaction towards external stimuli (example: telephone ringing - emotional outburst)			0.712^**^	
Obsession towards emotional event, although it has taken place long time ago (example: death of family member or friend)			0.575^**^	
Not interested in surroundings			0.655^**^	
Looking for support/assurance from partner			0.743^**^	
Night-day rhythm disruption			0.371^**^	
Restlessness at night				0.807^**^
Confusion after moving houses/in a new environment				0.674^**^
Outsiders aware of changes in term of behavior / appearance				0.675^**^

**P value was significant at <0.05 | The Pearson’s correlation coefficient is shown

### Reliability (internal consistency and test-retest)

Values for all items total correlation for concentration and sleep/environment was more than 0.4 (0.43–0.66) indicating that the items had moderate to good correlation with the other items comprising the overall score. Whereas the values for memory and physical/emotional symptoms were within the range of 0.29–0.62 indicating that the correlation between the items to the other items comprising the overall score was weak to moderate. The lowest score was 0.29 which was one item under memory scale.

The overall internal consistency was good with Cronbach’s alpha of 0.874. When broken into various subscales, the Cronbach’s alpha was above 0.7, which was acceptable for all scales – memory, concentration, physical and emotional symptoms and sleep and environment. The Cronbach’s alpha if each item were deleted one by one was above 0.8 for all questions. The item total correlation and Cronbach’s alpha coefficients for EDQ are shown in Table 6.

**Table 6:** Item total correlation and Cronbach’s alpha coefficients for EDQ

Scale	Item	Item total correlation	Cronbach alpha if item deleted	Cronbach’s alpha	Overall Cronbach’s alpha
Memory	Require check list as memory support	0.292	0.880	0.741	0.874
	Difficulty in remembering events that took place in the past 1 week (recent memory)	0.452	0.871		
	Unable to find kept item	0.514	0.867		
	Difficulty in remembering names/familiar faces	0.621	0.864		
	Difficulty in remembering familiar road directions	0.500	0.866		
	Repetitive questioning	0.530	0.865		
Concentration	Difficulty in following conversation	0.657	0.870	0.764	
	Difficulty understanding reading	0.554	0.862		
	Difficulty following stories on television	0.581	0.861		
Physical and Emotional symptoms	Difficulty carrying out daily house chores/work/hobby	0.486	0.869	0.754	
	Difficulty in taking care of personal hygiene or using the toilet	0.367	0.873		
	Disrupted movement (physical restlessness)	0.609	0.863		
	Unsuitable reaction towards external stimuli (example: telephone ringing - emotional outburst)	0.602	0.869		
	Obsession towards emotional event, although it has taken place long time ago (example: death of family member or friend)	0.376	0.872		
	Not interested in surroundings	0.517	0.868		
	Looking for support/assurance from partner	0.519	0.867		
Sleep and environment	Night-day rhythm disruption	0.594	0.869	0.720	
	Restlessness at night	0.642	0.867		
	Confusion after moving houses/in a new environment	0.426	0.869		
	Outsiders aware of changes in term of behavior/appearance	0.396	0.868		

Intraclass Correlation Coefficient (ICC) was done to assess the test retest reliability the first EDQ score and the retest EDQ score among those with MMSE of 21 and below. It ranged from 0.728–0.831 in all the components (Table 7). The overall ICC was an acceptable 0.764.

**Table 7:** Test retest Intra Class Correlation Coefficient (ICC) of the EDQ instrument (*N* = 160)

Component	Test – retest ICC (95% CI)		*P*-value
Memory	0.796	(0.732, 0.846)	<0.001
Concentration	0.831	(0.777, 0.873)	<0.001
Physical and Emotional symptoms	0.728	(0.647, 0.793)	<0.001
Sleep and Environment	0.811	(0.751, 0.858)	<0.001
Total (overall)	0.764	(0.538, 0.865)	<0.001

## Discussion

The term pseudo-dementia was developed by Kiloh (1961) to describe the cases that mimicked the picture of dementia especially depression [15]. Most researchers have suggested that the memory deficits in depression represent general cognitive inefficiency and attention problems rather than a fundamental lack of ability due to structural deficits. This can be seen in the differences between depression and dementia patients as well as in the temporariness of these deficits in the clinical picture of depression [16]. Many features overlap between depression and dementia and often exist together in older population making it difficult to determine the primary diagnosis [17]. However, for this study, we aim to exclude patients with depression as a reversible cause of pseudo-dementia but keeping in mind that these patients will need cognitive testing once treated and as part of their evaluation.

This study was done to validate the Early Dementia Questionnaire (EDQ) as a screening tool to detect patients with early dementia in primary care. Preliminary study of this new tool had shown that it is a simple, easy to administer and user friendly tool for screening of early dementia [12]. During this study, a few words were deleted from the original questionnaire (question 11 and 15) based by the response made from the pilot study of 50 respondents to make it clearer without changing the meaning of the questions. Question 11 was ‘difficulty in taking care of self/personal hygiene or using the toilet’. The word ‘self’ was deleted as the respondents felt that ‘personal hygiene’ was enough to understand the question. For question 15, the original was ‘apathy/no passion/not interested in surroundings’. The respondents found difficulty in understanding the word ‘apathy’ and ‘no passion’. Thus it was decided that the words were deleted and only ‘not interested in surroundings’ remained which did not change the meaning of the question.

Exploratory factor analysis (EFA) was done on all 20 symptoms. From the original 6 subdomains of EDQ, the symptoms were rearranged and regrouped into 4 subdomains without omitting any of the questions. The symptom ‘repetitive questioning’ from the subdomain concentration was noted to best fit into memory subdomain. This was supported by the literature that had stated ‘repetitive questioning’ was part of memory complaints in patient with early dementia [18–20]. Confirmatory factor analysis which was done on the 4 subdomains showed that all of the symptoms had factor loadings of more than 0.4 which showed good construct validity except the symptom ‘difficulty in taking care of personal hygiene or using the toilet’ with factor loading if 0.35. However, this was still acceptable. This question remain in the questionnaire as changes in ability or behavior are known to be part of the changes in patients with dementia [19, 21].

In item total correlation, while all of the other symptoms showed moderate to good correlation with the other items, one symptom; ‘require checklist as a memory support’ had the lowest score which was 0.292. This was rather unexpected. However, this question was not omitted as the Cronbach’s alpha for this subdomain was good (0.741). Furthermore, this symptom is also one of the memory complaints present in those with possible dementia [22, 23].

Cronbach’s alpha coefficients for each subdomains – memory (0.741), concentration (0.764), physical and emotional symptoms (0.754) and sleep and environment (0.720) showed that the internal consistencies of these subdomains were adequate. The overall Cronbach’s alpha for the whole EDQ was higher at 0.874. The Intraclass Correlation Coefficient between the first EDQ score and the retest EDQ score among those with MMSE of >21 showed a very strong overall agreement, ICC = 0.764, *N* = 160, *P* <0.001. This showed that the EDQ was a reliable tool in detecting early dementia.

There are some notations in the weakness in using Cronbach’s alpha. There are arguments that the alpha is not wholly a measure of internal consistency and should be measured with other parameters [24]. However, the alpha does not measure the degree of unidimensionality or multidimensionality but it just suggest. The alpha values reported in this study was satisfactory and showed the factorability of the factors.

Preliminary study on EDQ used 8 or more as the cutoff point to determine whether a patient had possible early dementia [12]. However, in this study a cutoff score of 10 is finally chosen after looking into the ROC curve and the sensitivity and specificity of different scoring. Thus, the sensitivity and specificity for EDQ was 71.2% and 59.5% respectively giving a PPV of 32.1% and NPV of 88.5%. The false positive rate of EDQ was 40.5%. However, high false positive rate is a characteristic of screening instruments used to detect diseases which has low prevalence rate [25].

In the study done by Folstein et al (1975) [26], the cut of point of the score for MMSE was 23 with the sensitivity of 100% and specificity of 44%. It is widely used in many other studies with the sensitivity ranging from 71.1–85.1% and specificity ranging from 81.3–95.6% depending on the study setting [27]. The original English version of MMSE has been used worldwide and since then has been translated and validated in various languages.

In this study, the validated Malay version of MMSE -7 (serial 7) with the cut off level of 21 and below was used to detect dementia. The sensitivity and specificity at this cut off were 88.5% and 75.3% respectively. Although EDQ had a lower sensitivity of 71.2% and specificity of 59.5%, it was still useful as a screening tool, with its advantages of being simple, easily administered and a user friendly tool. This is important especially in clinics where there were heavy clinic attendees and in rural clinics where not all health staff could be trained to use complicated screening tools.

However, being a new questionnaire, EDQ has its own limitations. The EDQ does not distinguish between patients with early dementia and mild cognitive impairment which may present with the same symptoms. This would rise to the high false positive rate. But a positive EDQ may alert the health care personnel to further assess the possibility of a patient having dementia.

EDQ is a patient and informant questionnaire. It is postulated that the information obtained from a patient would be more accurate if it is combined with the information taken from the informant. This is more important in patient who already have cognitive impairment which tend to overestimate their abilities and as the disease progresses, their self awareness of cognitive impairment also deteriorates [28].

There are other informant based questionnaire namely, the Informant Questionnaire on Cognitive Decline in the Elderly (IQCODE). It was originally described as a 26-item informant questionnaire to determine change in cognitive and functional performance retrospectively over a 10-year time period [29]. For each item, the scores change on a five-point ordinal scale, with responses ranging from 1: 'has become much better' to 5: 'has become much worse'. This gives a totel score of 26–130 that can be averaged by the total number of completed items to give a final score of 1.0–5.0. The higher scores indicate greater decline. A shortened 16-item version of IQCODE is available and commonly in clinical practice [30]. However, there are very limited research in the use of IQCODE in primary care settings which focus more on hospital and memory clinic settings [31].

The Quick Dementia Rating System (QDRS) is a new tool which consists of a 10-item questionnaire completed by an informant without the need for a trained clinician or rater. The scores range from 0–30 with higher scores representing greater cognitive impairment. QDRS has ten domains which are; (1) memory and recall, (2) orientation, (3) decision-making and problem-solving abilities, (4) activities outside the home, (5) function at home and hobbies, (6) toileting and personal hygiene, (7) behaviour and personality changes, (8) language and communication abilities, (9) mood, and (10) attention and concentration [32]. However, the QDRS was developed and validated in memory clinics where the prevalence of MCI and dementia is high and yet to have any studies in the community or primary practice.

### Limitations

In this study, the patient and informant were required to provide retrospective information, thus recall bias was unavoidable. Although in this study, there were no difficulties in having a reliable informant, it is foreseen that a reliable informants would not always be available. As not all of the respondents were able to converse well in English, there were some difficulties in understanding the questionnaire. However, as EDQ was an interviewer guided questionnaire, this problem was overcome when it was taken by the same trained interviewers to reduce the biasness.

The prevalence of dementia in this validation study were based on EDQ and MMSE only which serves as a screening tool. Dementia remains a clinical diagnosis, based on history from the patient and suitable caretakers and direct examination including cognitive assessment. Thus, further study would be to follow up with those patients who have possible dementia to document any changes or progression of the disease.

## Conclusion

This study showed that EDQ is a valid and reliable tool to be used as a screening tool to detect early dementia in primary care setting. The cut off score to determine possible early dementia in a patient is 10. EDQ could be used together with other established assessment tools to improve the detection of dementia. We suggest that EDQ to be used periodically once a patient is screened positive with EDQ to observe for future deterioration.

## Abbreviations

CFA: Confirmatory factor analysis
EDQ: Early Dementia Questionnaire
EFA: Exploratory factor analysis
ERGS: Exploratory Research Grant Scheme
GDS: Geriatric Depression Scale
ICC: Intraclass Correlation Coefficient
IQCODE: Informant Questionnaire on Cognitive Decline in the Elderly
IQR: interquartile range
MMSE: Mini Mental State Examination
NMRR: National Medical Research Register
NPV: negative predictive value
PPV: positive predictive value
QDRS: Quick Dementia Rating System
ROC: receiver-operating characteristic
SD: standard deviation
SPSS: Statistical Package for the Social Sciences
